# Adapting Sensory Analysis to the Pandemic Era: Exploring “Remote Home Tasting” of Sous-Vide Chicken Breast for Research Continuity

**DOI:** 10.3390/foods14040647

**Published:** 2025-02-14

**Authors:** Francesca Masino, Giuseppe Montevecchi, Andrea Antonelli, Domenico Pietro Lo Fiego, Patrizia Fava, Roberta Foligni, Andrea Pulvirenti

**Affiliations:** 1 BIOGEST-SITEIA Interdepartmental Center, Department of Life Sciences (Agro-Food Science Area), University of Modena and Reggio Emilia, Piazzale Europa 1A, 42124 Reggio Emilia, Italy; francesca.masino@unimore.it (F.M.); andrea.antonelli@unimore.it (A.A.); domenicopietro.lofiego@unimore.it (D.P.L.F.); patrizia.fava@unimore.it (P.F.); andrea.pulvirenti@unimore.it (A.P.); 2 Dipartimento di Scienze Agrarie, Alimentari ed Ambientali, Università Politecnica delle Marche, Via Brecce Bianche, 60131 Ancona, Italy; 3 Department of Human Sciences and Promoting of the Quality of Life, San Raffaele Telematic University Rome, Via Val Cannuta 247, 00166 Rome, Italy

**Keywords:** sous-vide, sensory attributes, randomized incomplete block, sensory evaluation card, olfactory characteristics, COVID-19 pandemic

## Abstract

Background: The pandemic and lockdown caused a slowdown or halt in many work activities across sectors, including academic research, which had to adapt lab procedures to lockdown restrictions. This study aimed to assess an innovative approach to sensory analysis that aligned with the pandemic’s constraints and could enhance traditional methods even in normal conditions. Methods: Remote training of judges was conducted to test the method’s effectiveness. Sensory evaluation of sous-vide chicken breast fillets was conducted at different temperatures (60, 70, 80 °C) and time combinations (60, 90, 120, 150 min), compared to a control (boiled at 100 °C for 60 min). Judges tasted 6 out of 13 randomized samples, recording intensities on a cloud-based sensory card. Results: Judges demonstrated good repeatability and panel homogeneity (RSD ≤ 30%). Significant differences (*p* < 0.05) in olfactory and flavor characteristics were noted among samples. Higher-temperature samples had stronger boiled meat and chicken flavors, and sous-vide samples showed greater juiciness, especially LT2 and LT3. Conclusions: The remote home-tasting approach proved effective in distinguishing key differences in meat characteristics based on cooking conditions. This method’s reliability and adaptability make it a promising alternative to lab-based sensory evaluation, ensuring research continuity in restrictive conditions and broadening potential for decentralized studies.

## 1. Introduction

In the last few years, the pandemic and the resulting lockdown caused a general slowdown, and in some cases even the total block, of many work activities in different sectors and at different levels [1,2,3]. Going to the workplace and meeting colleagues and collaborators could also contribute unconsciously to the spread of the virus.

Some areas of research have heavily been penalized. It was necessary to think about and develop measures to contain the spread of the virus and the infection, and intervention measures, developing plans and procedures to support them [4].

To satisfy the moment’s necessity and ensure continuity of activities, it has been necessary to find alternative solutions such as working “from home”, which combines telework, described as a way to work “away from the office” by employing electronic connections [5], and “smart working”. The latter can be regarded as an evolution of teleworking, but the adjective “smart” puts an emphasis on the potentially positive effects that this new modality is expected to bring about, for both companies and people. Smart working is “telework that individual perform in a smart and innovative manner” using devices for distant communication [1,6,7].

The complex organization and development of Internet Telecommunication (IT) systems and platforms, as well as the adaptation and enhancement of the entire IT system, has made it possible to develop a unique way of working. This new working approach is appreciated by many people who consider it convenient and practically compatible with their own needs and styles of daily life, even if, however, it is not without contraindications [2,4,6,8,9].

Regarding research centers, it was necessary for them to adapt their research methods, usually carried out on-site, to the restrictions imposed by the lockdown, and at the same time to implement new investigation strategies. Remote analysis approaches have been developed in clinical trials to optimize interviews, delivery of measuring devices, delivery of investigational drugs, measurements, and data collection [10], as well as in the field of online and remote learning [11]. A recent research study with promising results has been carried out in the food science sector [12]. Within the food science sector, sensory analysis is a scientific discipline that studies the perceived quality of food through an appropriately selected “panel of judges”, and enables objectively describing the sensory properties of consumer products by evaluating their intensity in both absolute terms and compared to other similar products [13,14]. To this end, some steps are involved, including meetings and tasting sessions conducted in a sensory analysis laboratory [15], in order to avoid possible psychological and physiological sensory errors [13].

The aim of this work was to develop and evaluate an innovative remote sensory analysis method designed to address the constraints posed by the pandemic. This study focused on the sensory evaluation of sous-vide chicken breast using a home-based sensory panel. By involving judges trained specifically on this food product, this research work aimed to assess the reliability, effectiveness, and potential scalability of this approach for both research and industrial applications.

## 2. Materials and Methods

### 2.1. Sample Collection and Preparation

Boneless chicken breast fillets were purchased from the local market and prepared immediately, as reported by Haghighi et al. [16]. In short, each piece of about 30 g, cut by hand with a knife, and measuring 4 cm × 5 cm with a thickness of 1 cm, was vacuum-sealed using a vacuum packaging machine (Elegen by Klefis Group s.r.l., Scandiano, Reggio Emilia, Italy) with a pump flow rate of 30 L to create a 98% vacuum degree inside the pouches. These were thermo-resistant plastic pouches (JoelPlas S.L., Barcelona, Spain) with the following characteristics: nylon-polyethylene, 150 mm × 200 mm, 80 μm thickness, thermal resistance from −40 °C to +120 °C, O_2_ permeability of 9 cm^3^/m^2^/day (4 °C, 80% relative humidity), and water vapor permeability of 1.2 cm^3^/m^2^/day.

The vacuum-sealed samples were cooked, as described by Haghighi et al. [16], by immersion in tap water in a sous-vide cooker (PFE-0065 Sous Cooker Professional 2000 watt, Elegen, Reggio Emilia, Italy) at different combinations of temperature (low temperature, LT, 60 °C; medium temperature, MT, 70 °C; high temperature, HT, 80 °C) and time (60; 90; 120; 150 min), at atmospheric pressure (Table 1).

A control sample (C) was prepared by sealing the chicken breast fillets in the same thermo-resistant plastic pouches without vacuum applied and cooked in tap water at 100 °C for 60 min. This procedure was implemented to avoid contamination of the product and to simulate the cooking of the other samples. After cooking was complete, the pouches were immediately cooled down to room temperature before further analysis. Samples were prepared in duplicate.

Moisture content, cooking loss, pH, lipid oxidation (Tbars), L* and a* color indices (CIELab coordinates by colorimeter, CR-400, Konica Minolta, Osaka, Japan), and shear force (Texture analyzer, Z1.0, Zwick/Roell, Ulm, Germany) were analyzed using the methods described by Haghighi et al. [16].

### 2.2. Preliminary Activities for Sensory Evaluation

Before carrying out the sensory evaluation, a survey was prepared using Google Forms and sent by email to all the judges (Figure S1). Questions were related to the judges’ personal information (age, gender), the frequency of meat and chicken consumption, the cooking methods most frequently used for meat preparation, and which sensory aspect should be most enhanced during meat taste, by choosing from the following list of attributes: appearance, tenderness-juiciness-chewiness, flavor, smell, and other attributes. The number of identical answers to the survey questions was percentage-based.

In addition, a preview of the sensory card was sent by email to all the judges to familiarize themselves with the terminology of the attributes, tasting procedure, and kind of scale used (Figure S2). Furthermore, this card described the guidelines on how to store and prepare the sample at home.

Each sample enclosed in the vacuum-sealed plastic bag was anonymized using a random three-digit numerical code and subsequently placed inside a food container and delivered to the judges’ homes one day before the sensory evaluation.

Standard samples of “steamed chicken”, samples of “meat cooked at different degrees of doneness”, and aqueous solutions of caffeine, citric acid, ferric sulfate, and tannin were also added to the food container. In addition, the judges were asked that the samples and standards be stored on the top shelf of the refrigerator at 4 °C.

The day before the sensory session, the panel leader checked the online connection with all the judges and, during that meeting, of a 2 h duration, he ascertained that all the instructions of sensory evaluation (clarity and definitions of attributes, methods of tasting samples, and methods of evaluation of attributes) and preparation of samples had been assimilated. In addition, judges were provided with detailed guidelines in accordance with ISO 8589:2007 [17] and the recommendations described in MEBAK [18] and Meilgaard et al. [13]. Judges were instructed to conduct sensory evaluation in quiet and isolated environments at room temperature, with natural or neutral lighting to avoid color distortions, and to have minimal external distractions such as noise, odors, or personal fragrances. They were also advised to avoid consuming drinks or coffee or smoking before the session. Specific instructions for sample preparation, including warming and serving conditions, were given to reduce variability.

A training update was conducted. The judges were instructed to perceive the SBM, FBM, SMC, and FMC attributes using “steamed chicken” samples that were heated though a microwave oven under the same conditions (30 sec at 600 W) as the samples. These samples were also used to define the texture attributes by the judges. Furthermore, samples of “meat cooked at different degrees of doneness” were used to anchor them to the 10-point scale relative to doneness.

“Other perceptions” (OP) was meant as anomalous perceptions, characterized by bitter, acidic, metallic, or astringent notes, or a combination of them. During training sessions, standard aqueous solutions at a concentration of 0.1 g/L (caffeine), 0.5 g/L (citric acid), 0.01 g/L (ferric sulfate), and 0.5 g/L (tannin) were used by the judges to familiarize themselves with each taste in order to identify them in the samples. The judges measured the OP intensity in relation to the standard aqueous solution concentrations.

### 2.3. Sensory Evaluation Card

The sensory evaluation card (created using a cloud version of the Smart Sensory Box software v2.12.9 (Smart Sensory Solution S.r.l., Sassari, Italy) included the assessment of visual, smell, flavor, and texture attributes. Table 2 shows the list of the 12 sensory attributes selected for sample evaluation, their definition, and the testing procedure (sample amount to test, time that the product should remain in the mouth, and, regarding the smell evaluation, the distance of the sample from the nose).

Before the evaluation, each judge received by mail the link to access the sensory evaluation cards of the samples that they had to evaluate in a specific order.

### 2.4. Sensory Evaluation and Home Tasting

Thirteen judges (J1–J13) (men and women, aged between 25 and 60) participated as volunteers in the sensory evaluation of the samples. All the judges were expert meat tasters because they had already been trained [19] on the sensory attributes of the specific product. Therefore, the reliability of the judges was ensured by previous participation in other sensory studies on meat. However, an update of the training was conducted as described in Section 2.2.

The remote descriptive sensory evaluation of the samples was conducted following a QDA methodology [14]. On the day of the sensory session, all the judges connected at the same time (3.30 p.m.) via Google Meet. Each judge prepared the samples in the assigned order and one at a time. The samples were individually prepared by heating them in a microwave oven (30 s at 600 W), then removed from the package and tasted respecting all the precautions described in Section 2.2 to avoid distractions [13]. All the panelists remained connected for the duration of the tasting session, keeping the cameras on.

Each judge evaluated constant quantities of each sample (30 g), and waited 1 min between tasting consecutive samples, during which he/she drank water and ate pieces of unsalted crackers. During the analysis, the judges smelled and tasted the samples and rated the intensity of each sensory attribute on a 10-point scale (numeric rating scale) [20]. The score “1” indicated that the attribute was perceived as very low, while “10” indicated a very high intensity of perception [13,21].

The panel leader supervised the entire duration of the sensory assessment, remaining connected and available to facilitate the judges’ work.

When filling out the online evaluation form, the judges’ responses were automatically saved in a spreadsheet used for data analysis. Each judge tasted a total of 6 samples (4 different samples out of the 13 studied + 2 replicas of 2 of them) according to an incomplete randomized block design [13,22,23]. In this way, each meat sample was evaluated a total of 6 times by 4 different judges. The judges evaluated the attributes of the meat in the order listed on the sensory card (Figure S2). Different sections were outlined and presented in the following order: visual attributes (D), aroma (SBM and SMC), taste sensations (FBM and FMC, OP), and texture (TEND-FB, TEND, JUIC, JUIC-F, RC, and CHEW).

### 2.5. Statistical Analysis

The judges’ performances were evaluated in terms of homogeneity and repeatability using the relative standard deviation (RSD) and the box-and-whisker plot. In addition, analysis of the variance (one-way ANOVA) was conducted among samples. The differences among sample mean values were compared using Tukey’s post hoc test (*p* < 0.05).

Pearson coefficients of correlations (r) among all sensory and physico-chemical attributes (Table 3 of Haghighi et al. [16]) were calculated.

Principal component analysis (PCA) was carried out on the matrix of the autoscaled data, including both the results of the sensory analysis (Table 3 of the present manuscript) and the physico-chemical data of each sample (taken from Haghighi et al. [16]; Table 3. Moisture Content, Cooking Loss, Shear Force, TBARS, Color Parameters—L*: Lightness, a*: Redness/Greenness, and b*: Yellowness/Blueness, and pH of Sous vide Chicken). Breast Fillets Cooked at Different Temperature and Time Combinations). Testing was performed using Statistica v8.0 software (previously developed by Stat Soft Inc., now TIBCO Software Inc., Palo Alto, CA, USA).

## 3. Results

### 3.1. Results of the Survey

Regarding the results of the survey carried out on judges, the sample group was composed of eight women and five men; two judges aged between 18 and 25, two judges aged between 26 and 35, three judges aged between 36 and 45, five judges aged between 46 and 55, and one judge over 60 years old. All judges were experienced in sensory analysis, having undergone extensive training that included sessions to recognize sensory attributes such as tastes, aroma, flavor, and texture. In addition, they participated in tests with standardized samples to calibrate their sensitivity, consistency, and accuracy.

**Figure 1:**
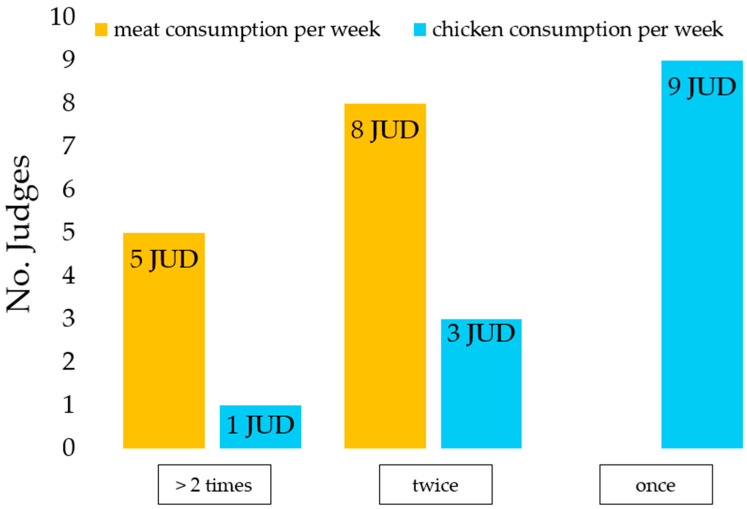
Results of the survey on meat consumption and chicken consumption per week.

Figure 1 shows that all the judges reported consumption of meat, of at least twice a week for 62% of them (8/13 judges), while chicken was consumed at least once a week by 69% (9/13 judges) of respondents. In addition, the highest percentage of judges (69%) identified tenderness, juiciness, and chewing as the most relevant sensory properties (9/13 judges).

### 3.2. Judges’ Performance

**Figure 2:**
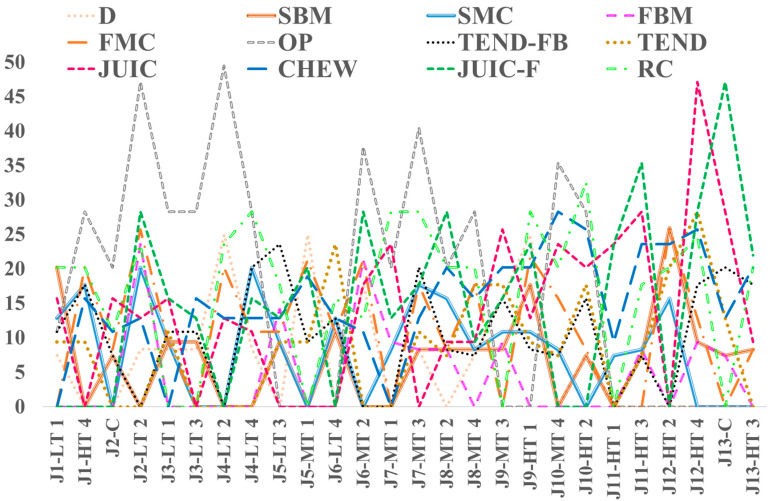
RSD values calculated between ratings provided by the same judge for the same sample for each attribute. J1, J2,…, Jn represent the judges, while alphanumeric codes identify the evaluated samples. LT1, MT1, HT1, LT2, MT2, HT2, LT3, MT3, HT3, LT4, MT4, HT4: samples prepared using low (L), middle (M), or high (H) temperature (60, 70, 80 °C, respectively); for 60 (1), 90 (2), 120 (3), 150 (4) min, respectively. C: control sample. D: Doneness; SBM and FBM: Smell and Flavor cooked meat; SMC and FMC: Smell and Flavor meat-like cooked chicken; OP: other perceptions; TEND-FB: Tenderness at first bite; TEND and JUIC: Tenderness and Juiciness after 5 chews; JUIC-F: final juiciness when the meat is ready for swallowing; CHEW: chewiness; RC: residues of chewing.

Figure 2 shows the repeatability of the judges. Relative standard deviations (RSDs) were calculated for each judge and for each attribute, using the average value of the scores obtained by each judge in the repeated evaluation of a single sample, while a threshold value (RSD = 30%) was arbitrarily set.

A good repeatability of the judges (RSD ≤ 30%) was noted at a general level. However, some judges showed higher values for some attributes. The “other perceptions” (OP) attribute showed an RSD higher than 30% for five judges, probably because it was not related to a single and defined sensory characteristic, and therefore its evaluation was fairly subjective. In addition, “juiciness” (JUIC) and “final juiciness” (JUIC-F) showed RSD higher than 30% for 1 and 2 judges, respectively. The final juiciness was defined as the juice amount perceived when the meat is ready for swallowing. In fact, it can also be influenced by the judge’s chewing concerning the quantity of sample or the portion of meat tasted (for example, if fibrous), or even to the tasting conditions. Based on the preliminary sessions conducted with the judges involved in the study, the main cause of variability in the evaluation of this attribute, observed for example in judges J11 (HT3) and J13 (C), could be related to the texture of the sample. The use of a more representative sample could improve the results of the repeatability of the judges’ evaluation during the tasting of the same sample.

**Figure 3:**
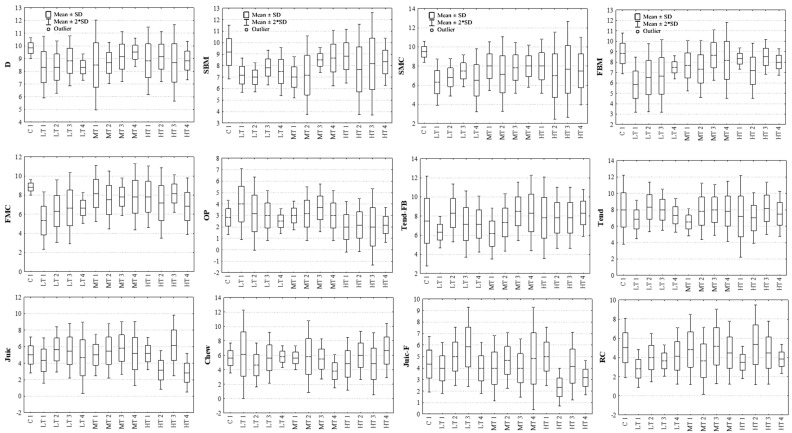
Box-and-whisker plot of the samples grouped by variable. Boxes represent mean value ± SD (standard deviation), whiskers are the mean value ± 2 × SD. LT1, MT1, HT1; LT2, MT2, HT2; LT3, MT3, HT3; LT4, MT4, HT4: samples prepared using low (L), middle (M), or high (H) temperature (60, 70, 80 °C, respectively); for 60 (1), 90 (2), 120 (3), 150 (4) min, respectively. D: Doneness; SBM and FBM: Smell and Flavor cooked meat; SMC and FMC: Smell and Flavor meat-like cooked chicken; OP: other perceptions; TEND-FB: Tenderness at first bite; TEND and JUIC: Tenderness and Juiciness after 5 chews; JUIC-F: final juiciness when the meat is ready for swallowing; CHEW: chewiness; RC: residues of chewing.

The performance of the judges was also investigated through the box-and-whisker plot of the samples grouped by attribute (Figure 3). The data showed modest dispersion, thus indicating general agreement among judges for most attributes. The results obtained for D, SMC, FMB, FMC, TEND-FB, TEND, and CHEW attributes in the samples C, LT1, LT4, MT1, and MT4 were an example of this agreement. In addition, no “outlier” judges were found for any attribute evaluated in the different samples.

Regarding JUIC-F and CR, the higher dispersion of results indicated lower panel homogeneity in samples C, MT1, MT3, MT4, and HT2. Notably, these outcomes could not be attributed to specific cases or individual judges. The situation was further complicated by the use of an incomplete randomized design for sample evaluation. Moreover, enhancing the judges’ awareness is essential to achieving more consistent results.

In this context, one potential factor contributing to variability is the remote sensory evaluation setting. While this approach offers several advantages, uncontrolled home environments might impact the reliability of the results. The variability of the conditions under which the tests take place, such as differences in lighting, noise levels, and temperatures in the judges’ homes, might affect their sensory perception. Providing judges with more detailed guidelines on setting up a standardized testing environment and supplying standardized tools (e.g., uniform white plates, standard lighting equipment and appropriate position of the light source, active noise-canceling earphones to aid concentration, and temperature- and humidity-control devices) could help further reduce variability.

### 3.3. Sensory Evaluation of Sample

Table 3 shows the values of sensory evaluation. Some parameters, such as doneness, other perceptions, tenderness at first bite, tenderness, rests of chewing, and chewiness, did not show significant differences among the samples. Conversely, significant differences were shown regarding all smell and flavor attributes (meat boiled smell and flavor, meat like-chicken cooked smell and flavor). The sample C showed the highest values of these attributes, and the differences from this sample decreased when temperature and time cooking increased in sous-vide samples.

Regarding the texture parameters (JUIC and JUIC-F), the significant differences were more interesting. The LT2 samples for JUIC (5.67) and LT3 for JUIC-F (5.83) showed greater juiciness than C, while the same parameters decreased as the temperature and cooking time increased. It is likely that sous-vide conditions influenced the higher consistency rating [24].

Regarding the effects of sous-vide cooking method on flavor, no agreement has been found in the literature [24,25], while, regarding the texture, there is greater agreement. In particular, meat cooked with low-temperature methods showed greater juiciness and tenderness than conventional cooking methods (boiling, baking, grilling) [24,26,27]. However, sensory results are highly dependent on temperature/time combination, meat type, and quality [28,29].

Regarding olfactory parameters, LT samples (1–4) were less odorous and with the lowest values compared to samples obtained at higher temperatures. For all these four attributes (SBM, FBM, SMC, and FMC), a trend was observed with the increase in temperature applied in sous-vide cooking. In fact, the HT samples showed higher values of “meat boiled smell and flavor” and “meat like-chicken cooked smell and flavor” compared to the MT or LT samples, and this trend was confirmed as the cooking time increased.

Although sous-vide cooking is known to reduce the loss of volatile compounds of the smell and taste of meat [30,31], the C samples always showed higher values for the olfactory attributes than the sous-vide samples.

According to Shi et al. [32], some volatile compounds were generated in cooked meat through the degradation and oxidation of lipids. Hexanal and 2,4-decadienal were the most abundant aldehydes identified in chicken flavoring and were known to be the major oxidation products of linoleic acid. Both the aldehydes along with 2-methyl-3-furanthiol were generated by the Maillard reaction. The latter is the most important volatile compound responsible for the “meaty” flavor of chicken broth [33]. However, these volatiles tend to get lost quickly if the meat samples are not packaged.

This would explain the reason why C samples maintained high intensities of volatile compounds. In fact, in C samples, an increase in lipid oxidation is likely to have occurred due to the high temperature (100 °C for 60 min), along with the presence of oxygen in the pouches, which, in turn, caused the formation of a mixture of compounds that positively affected the odor. Since the C samples were closed in plastic bags to preserve contamination and to simulate the sous-vide cooking procedure of the other samples, the “lost water”, in this case, accumulated in the bags, remaining in contact with the sample for the entire storage time before its sensory evaluation. It is possible that the close contact between the lost cooking water and the meat, as well as the saturated environment in the package, positively influenced the smell and taste evaluation of the control (C samples).

### 3.4. Linear Correlation Evaluation

In a previous study, chemical analyses such as moisture content (MOI), cooking loss (CL), pH, color, thiobarbituric acid reactive substances (TBARS) and shear force (SF) were carried out on the same sample set [13]. Table 4 shows all the significant coefficients of correlation (*p* < 0.05) obtained among chemical parameters and sensory attributes. It is worth mentioning a negative correlation between the smell and flavor of samples (SMC, FBM, SBM) and the MOI values (−0.63, −0.67, and −0.73, respectively) and a positive correlation between FMC, SMC, SBM, FBM, and CL values (0.68, 0.69, 0.73, and 0.76, respectively). These results were consistent with each other and highlighted a possible dilution effect on the perception of odorous compounds. Different studies [16,24,30,34] have reported that sous-vide treatment showed the lowest cooking loss compared to conventional cooking, and it was increased by increasing cooking temperature and time. On the other hand, the volatile aromatic compounds responsible for the smell and flavor of the meat commonly develop at temperatures above 70 °C [33,35], which is when a higher cooking loss is observed. In addition, a positive correlation between smell and taste with the Tbars index was also shown (Table 4).

A significant inverse correlation between OP (bitter, acidic, metallic, and astringent perceptions) and pH (r = −0.57) was highlighted. The judges found the presence of acidic (15% of citations), bitter (15% of mentions), and metallic (7% of citations) notes. MT3 and LT1 were the samples where the sensation of acidic and bitter were mentioned the most times, and the latter sample received the most mentions of acidity overall. Christensen et al. [36] highlighted the presence of metallic and sour tastes in pork and chicken treated at a low temperature for a long time (LTLT). It is known that there is an inverse correlation between acidity and pH. In addition, although the literature is often contradictory on the subject, potentiating or suppressing effects have been reported between gustatory stimuli. Mixtures of acidic and bitter compounds, for example, enhanced each other at low intensity/concentration [37].

### 3.5. Principal Component Analysis (PCA)

**Figure 4:**
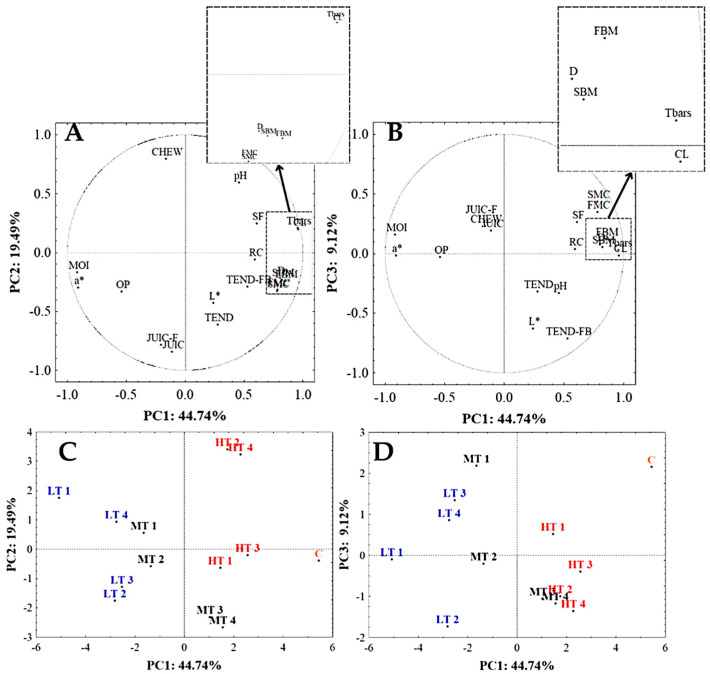
Loading plots of PC1 vs. PC2 (**A**) and PC1 vs. PC3 (**B**) and score plot PC1 vs. PC2 (**C**) and PC1 vs. PC3 (**D**) of the principal component analysis (PCA) of the data set. L* (luminosity), a* reddish, MOI (moisture), SF (shear force), Tbars (oxidation lipid index), pH, CL (cooking loss), D (Doneness), SBM and FBM (Smell and flavor cooked meat), SMC and FMC (Smell and flavor meat like chicken cooked) OP (others perceptions), TEND-FB (tenderness at first bite), TEND and JUIC (tenderness and juiciness after 5 chews), JUIC-F (final juiciness when the meat is ready for swallowing), CHEW (chewiness), RC (residues of chewing). LT1, MT1, HT1; LT2, MT2, HT2; LT3, MT3, HT3; LT4, MT4, HT4: samples prepared using low (L), middle (M), or high (H) temperature (60, 70, 80 °C, respectively); for 60 (1), 90 (2), 120 (3), 150 (4) min, respectively.

PCA was performed on a data set including sensory analysis data and data from a previous chemical evaluation study of the same samples [16]. More than 70% of the total variability (73.35%) was explained by the first three principal components (PCs). Figure 4A and 4B highlight the loading values in the spaces PC1 vs. PC2, and PC1 vs. PC3, respectively, suggesting that 13 variables (loading value ≥ |0.70|) explain most of the variability among the samples (a*, MOI, Tbars, CL, D, OBM, OMC, FBM, FMC, Tend-FB, JUIC, Chew, JUIC-F).

The PC1 vs. PC2 score plot (Figure 4C) showed that the distribution of samples was based on cooking temperatures. Going from left to right of the plot, samples are placed in the following order: LT (sous-vide at 60 °C), MT (sous-vide at 70 °C), HT (sous-vide at 80 °C), and last, C (100 °C). Regarding the MT samples, those with a shorter cooking time (MT1 and MT2) were placed close to the LT samples, while the MT3 and MT4 samples were grouped together with the HT samples. The different arrangements among samples were due to smell and flavor attributes, along with some parameters determined in the previous study [16], such as cooking loss (CL) and Tbars with positive weight on PC1. On the contrary, moisture content (MOI) and a* values were loaded onto PC1 with negative weight.

Most sensory attributes (smell and flavor perception), as well as some parameters (CL, Tbars, and SF), were higher in C and HT sous-vide samples and decreased in LT samples. Conversely, the latter samples were characterized by higher MOI and a* values. The reduced water loss during low-temperature cooking (especially LT2 and LT3) kept the samples moist. In addition, the juice influenced the consistency of the samples by improving juiciness (JUIC) and reducing shear force (SF). According to Ji et al. and Przybylski et al. [38,39], the juiciness of meat and meat products was improved by the sous-vide processing method.

MT3 and MT4 were judged as the most tender followed by HT1 and HT3 samples. The effects of the higher cooking temperature are close to those of the intermediate temperature for longer times, positively affecting the perception of tenderness of the meat. Both the parameters of tenderness showed an inverse correlation with chewiness, meant as “difficulty in reaching a swallowable bolus”.

Changes in meat tenderness during heat treatment are associated with two opposing heat-induced phenomena. While heat increases collagen solubilization, thus increasing tenderness, it also causes protein denaturation and meat shrinkage, thus decreasing tenderness [40]. According to Wright et al. [41], the complete conversion of collagen to gelatin can be achieved by increasing the cooking time or cooking temperatures. Since white meat is very rich in collagen, changes in textual properties depend heavily on these parameters. Previous studies [25,31] found that the tenderness of chicken breast fillets at low temperatures increased with sous-vide cooking time.

Tbars was positively correlated with water loss (CL) and the attributes related to the perception of odorous substances. Higher temperatures of cooking cause the formation of several volatile compounds, generated by the degradation and oxidation of lipids, with a generally pleasant effect on the smell and flavor of meat [32]. In addition, in conventional cooking of meat, the higher the temperatures, the greater the contribution of the Maillard reaction, providing meat for the characteristic flavor and appearance. For this reason, effective superficial browning of meat prepared with the sous-vide technique is required to make the product more palatable [42].

PC2 (19.49% of the total variability) shows a sample distribution based mainly on juiciness attributes (with negative weights) and chewiness parameters (with a positive sign) (Figure 4C). JUIC and JUIC-F showed an inverse correlation with pH, indicating that high juiciness led to a lowering of pH. Lower pH values were observed in LT2 and LT3. It is likely that this result may be attributable to the lower cooking temperatures used for these samples [16]. In fact, Becker et al. [43] reported that, on the contrary, the increase in temperature caused an increase in pH.

Samples HT2 and HT4 were segregated in the upper right part of the plot (Figure 4C) due to the high CHEW attribute and low values of JUIC and JUIC_F, as well as TEND.

Figure 4B shows the score plot of PC1 vs. PC3 (9.12 of the total variability), which allows better appreciation of the difference between the sous-vide samples cooked for 60 min and those cooked for 90 min, and those cooked for 120 and 150 min. The most influential parameter on PC3 was TEND-FB, which allowed MT3 and MT4, and HT2, HT3, and HT4, to be grouped together (Figure 4D).

## 4. Conclusions

This study demonstrates the feasibility of remote sensory evaluation as a reliable alternative to standard methods, particularly during major disruptions such as pandemics. While home tasting introduces potential environmental variability, the implementation of strategies such as remote training, standardized sample preparation, and guidelines for home environments allowed judges to effectively evaluate sous-vide cooked chicken. The results demonstrated good performance in terms of repeatability and homogeneity (RSD ≤ 30%) and significant product discrimination. Furthermore, the experimental data confirmed the impact of sous-vide cooking on sensory attributes, showing that low temperatures preserved tenderness and juiciness, while higher temperatures enhanced flavors.

Despite the promising potential shown by the data obtained, this approach still presents challenges, such as ensuring a complete standardization of conditions where tests are carried out and robust data validation. Further research is needed to enhance its reliability, including the development of advanced digital devices to standardize environmental parameters.

The results of this study suggest that remote home-tasting evaluation could be an intriguing area for further exploration in the food industry. Based on these findings, future research should investigate its potential for enabling decentralized assessments, allowing companies to maintain operations when access to centralized facilities is restricted. In addition, studies could explore the role of remote home tasting in expanding the geographic diversity of judges for more representative data, decreasing logistical costs by reducing the need for physical testing sites, and integrating the method into consumer testing.

## Figures and Tables

**Table 1:** Temperature and time conditions applied for the samples’ preparation through sous-vide cooking.

SAMPLES	TEMPERATURE (°C)	TIME (min)	NO. TASTING SAMPLES
C	100	60	6
LT 1	60	60	6
LT 2	60	90	6
LT 3	60	120	6
LT 4	60	150	6
MT 1	70	60	6
MT 2	70	90	6
MT 3	70	120	6
MT 4	70	150	6
HT 1	80	60	6
HT 2	80	90	6
HT 3	80	120	6
HT 4	80	150	6
Total	-	-	78

LT1, MT1, HT1, LT2, MT2, HT2, LT3, MT3, HT3, LT4, MT4, HT4: samples prepared using low (L), middle (M), or high (H) temperature (60, 70, 80 °C, respectively); for 60 (1), 90 (2), 120 (3), 150 (4) min, respectively. C: control sample.

**Table 2:** Sensory vocabulary, definitions, and testing procedures.

	Attributes	Definition	Testing Procedure
1	Doneness	Visual sensation of degree of cooking (D).	Evaluate the sample under white light. It goes from pink or light pink (when it is little or no cooked) to white (when it is well cooked).
2–5	Meat boiled smell and flavor Meat like-chicken cooked smell and flavor	Olfactory and flavor sensations that remember the smell and flavor of the boiled meat (SBM and FBM), and the meat like-chicken cooked (SMC and FMC) perceived with the nose and in the mouth.	Place the sample under the nose (1 cm) and broken it. Take 2–3 inhalations. Then, bring the sample to the mouth and chew it five times. Rate the intensity in terms of boiled meat smell and flavor and meat like-chicken cooked smell and flavor.
6	Other perceptions	Perceptions such as bitter, acidic, metallic, and astringent (OP).	Bring the sample to the mouth, chew 5 times, and rate other perceptions of the cooked chicken meat.
7	Tenderness at first bite	Texture sensation at first bite (TEND-FB).	Bring the sample between the teeth and evaluate the stress required to cut the meat.
8–9	Tenderness and Juiciness	Texture sensation after 5 chews (TEND and JUIC).	Bring the sample in mouth, chew 5 times, and rate the chewing easiness of the sample and the juice amount perceived.
10	Residues of chewing	Meat amount left when it is ready for swallowing (RC).	Bring the sample in mouth and evaluate the meat amount left when it is ready for swallowing.
11	Final juiciness	Texture sensation at end of chewing (JUIC-F).	Bring the sample in mouth chewing until is ready for swallowing and evaluate juice amount perceived. It is evaluating after “*Rests of chewing*”.
12	Chewiness	Texture sensation at end of chewing (CHEW).	Bring the sample in mouth and evaluate number of chews to make the sample ready at the swallowing. A low number of chews (1–3) corresponds to a very chewable sample, a middle number of chews (4–6) corresponds to sample mildly chewable, a high number of chews (7–10) corresponds to sample hardly chewable.

**Table 3:** Mean of sensory data for each sample for attribute ± SD (standard deviation).

Samples	C 1	±SD	LT 1	±SD	LT 2	±SD	LT 3	±SD	LT 4	±SD	MT 1	±SD	MT 2	±SD
D	9.83	0.41	8.33	1.21	8.33	1.03	8.83	0.98	8.33	0.52	8.50	1.76	8.67	0.82
SBM	9.17 ^a^	1.17	7.17 ^b^	0.75	7.00 ^b^	0.63	7.83 ^ab^	0.75	7.50 ^ab^	1.05	7.00 ^b^	0.89	7.17 ^ab^	1.72
SMC	9.50 ^a^	0.55	6.33 ^b^	1.21	6.83 ^b^	0.98	7.50 ^b^	0.84	6.50 ^b^	1.64	8.00 ^ab^	1.26	7.17 ^ab^	1.94
FBM	8.83 ^a^	0.98	5.83 ^b^	1.33	6.50 ^ab^	1.64	6.67 ^ab^	1.89	7.50 ^ab^	0.55	7.67 ^ab^	1.21	7.33 ^ab^	1.34
FMC	8.83 ^a^	0.41	5.33 ^b^	1.97	6.33 ^ab^	1.47	6.67 ^ab^	1.63	6.67 ^ab^	0.82	8.17 ^a^	1.07	7.50 ^ab^	1.49
OP	2.83	0.75	4.00	2.37	3.17	1.66	3.00	1.10	2.50	0.84	3.00	0.63	3.17	1.15
TEND-FB	7.50	3.15	6.33	0.82	8.33	1.51	7.17	1.72	7.17	1.75	6.17	1.33	7.33	1.51
TEND	8.00	2.10	6.83	1.17	8.33	1.51	8.00	1.26	7.33	1.63	6.50	0.84	7.83	1.72
JUIC	5.00 ^ab^	2.61	4.33 ^ab^	1.37	5.67 ^a^	1.37	5.50 ^ab^	1.64	4.67 ^abc^	1.49	5.00 ^abc^	1.26	5.50 ^ab^	1.67
CHEW	5.67	1.03	6.17	3.06	4.67	1.39	5.67	1.66	5.83	0.75	5.67	0.72	5.83	2.14
JUIC-F	4.33 ^ab^	1.86	4.00 ^ab^	1.21	5.00 ^ab^	1.26	5.83 ^a^	1.72	4.00 ^ab^	1.21	4.00 ^ab^	1.41	4.67 ^ab^	1.21
RC	5.00	1.55	2.83	0.98	4.00	1.82	3.67	0.61	4.17	1.72	4.83	2.14	4.00	1.75
**Sample**	**MT 3**	**±SD**	**MT 4**	**±SD**	**HT 1**	**±SD**	**HT 2**	**±SD**	**HT 3**	**±SD**	**HT 4**	**±SD**	**F Value**	***p* Value**
D	9.17	0.98	9.50	0.55	8.83	1.33	9.17	0.98	8.67	1.51	8.83	0.75	1.15	ns
SBM	8.50 ^ab^	0.55	8.67 ^ab^	1.21	8.83 ^ab^	1.17	7.67 ^ab^	1.99	8.17 ^ab^	2.23	8.33 ^ab^	1.03	2.05	*
SMC	7.83 ^ab^	1.33	8.00 ^ab^	1.10	8.00 ^ab^	1.41	7.00 ^ab^	2.28	7.67 ^ab^	2.50	7.50 ^ab^	1.76	1.69	*
FBM	8.67 ^a^	1.21	8.17 ^ab^	1.83	8.33 ^a^	0.52	7.17 ^ab^	1.33	8.50 ^a^	0.84	8.00 ^ab^	0.63	3.15	***
FMC	7.83 ^ab^	0.98	7.83 ^ab^	1.72	7.83 ^ab^	1.44	7.17 ^ab^	1.81	8.17 ^b^	0.98	6.83 ^ab^	1.47	2.71	**
OP	3.67	0.75	3.00	1.10	2.00	0.91	2.17	1.03	2.00	1.55	2.33	1.21	1.47	ns
TEND-FB	8.50	1.52	8.33	1.97	7.83	2.14	7.83	1.60	7.83	1.60	8.33	1.21	1.08	ns
TEND	8.00	1.55	7.83	1.83	7.17	2.48	7.00	1.79	8.17	1.60	7.50	1.38	0.71	ns
JUIC	5.53 ^ab^	1.64	5.17 ^abc^	2.07	5.17 ^abc^	0.98	3.17 ^bc^	1.17	6.17 ^a^	1.83	2.83 ^b^	1.17	1.82	*
CHEW	5.17	1.478	3.83	1.14	4.83	1.45	6.00	1.77	4.83	2.14	6.67	2.02	1.16	ns
JUIC-F	4.00 ^ab^	1.14	4.83 ^ab^	2.23	5.00 ^ab^	1.48	2.33 ^b^	1,51	4.17 ^ab^	1.22	3.17 ^ab^	0.75	2.25	*
RC	5.17	2.32	4.50	2.17	3.50	1.10	5.33	2.58	4.50	1.86	3.83	0.60	1.05	ns

LT1, MT1, HT1; LT2, MT2, HT2; LT3, MT3, HT3; LT4, MT4, HT4: samples prepared using low (L), middle (M), or high (H) temperature (60, 70, 80 °C, respectively); for 60 (1), 90 (2), 120 (3), 150 (4) min, respectively. D: Doneness; SBM and FBM: Smell and Flavor cooked meat; SMC and FMC: Smell and Flavor meat-like cooked chicken; OP: other perceptions; TEND-FB: Tenderness at first bite; TEND and JUIC: Tenderness and Juiciness after 5 chews; JUIC-F: final juiciness when the meat is ready for swallowing; CHEW: chewiness; RC: residues of chewing. The one-way ANOVA results are expressed as F values and *p* values (* ≤ 0.05; ** ≤ 0.01; *** ≤ 0.001; ns, not significant). Post hoc Tukey’s test (*p* value ≤ 0.05) is reported using different letters indicating that label average values that are significantly different (a > b > c).

**Table 4:** Significant coefficients of correlation (*p* ≤ 0.05).

	L*	a*	MOI	SF	Tbars	pH	CL	D	SBM	SMC	FBM	FMC	OP	TEND-FB	TEND	JUIC	CHEW	JUIC-F	RC
L*																			
a*																			
MOI		0.83																	
SF			−0.70																
Tbars		−0.96	−0.88	0.60															
pH		−0.65	−0.62		0.60														
CL		−0.92	−0.96	0.65	0.95	0.59													
D		−0.62	−0.71		0.65		0.75												
SBM		−0.71	−0.73		0.70		0.73	0.80											
SMC		−0.58	−0.63	0.56	0.68		0.69	0.78	0.74										
FBM		−0.73	−0.67		0.79		0.76	0.63	0.78	0.76									
FMC		−0.63			0.74		0.68	0.64	0.59	0.86	0.88								
OP		0.76			−0.69	−0.57													
TEND-FB	0.58																		
TEND														0.64					
JUIC																			
CHEW																−0.63			
JUIC-F						−0.55										0.70			
RC								0.57			0.57	0.68							

L* (luminosity), a* color value, MOI (moisture content), SF (shear force), Tbars (oxidation lipid index), pH, CL (cooking loss), D (Doneness), SBM and FBM (Smell and flavor cooked meat), SMC and FMC (Smell and flavor meat like chicken cooked) OP (other perceptions), TEND-FB (Tenderness at first bite), TEND and JUIC (Tenderness and juiciness after 5 chews), JUIC-F (final juiciness when the meat is ready for swallowing), CHEW (chewiness), RC (residues of chewing).

## Data Availability

The original contributions presented in the study are included in the article/supplementary material, further inquiries can be directed to the corresponding authors.

## Supplementary Materials

The following supporting information can be downloaded at: https://www.mdpi.com/article/10.3390/foods14040647/s1. Figure S1. Survey questionnaire sent to judges. The survey collects personal information, details on the frequency of meat and chicken consumption, preferred cooking methods for meat preparation, and opinions on which sensory attributes should be most enhanced during meat tasting (i.e., appearance, tenderness-juiciness-chewiness, flavor, smell, and other attributes). Figure S2. Sample sensory evaluation card sent to judges. This card introduces the terminology of sensory attributes, explains the tasting procedure, and provides guidance on the type of scale used for assessment.
