# Supplementary material for: Adapting Sensory Analysis to the Pandemic Era: Exploring “Remote Home Tasting” of Sous-Vide Chicken Breast for Research Continuity
Source: Foods. 2025 Feb 14;14(4):647. doi: 10.3390/foods14040647 (PMC11854824; doi:10.3390/foods14040647)
Supplement: Supplementary file 1 [file foods-14-00647-s001.zip › foods-3434735-supplementary.pdf]

Figure S1. Survey questionnaire sent to judges. The survey collects personal information, details on the frequency of meat and chicken consumption, preferred cooking methods for meat preparation, and opinions on which sensory attributes should be most enhanced during meat tasting (i.e., appearance, tenderness-juiciness-chewiness, flavor, smell, and other attributes).

Figure S2. Sample sensory evaluation card sent to judges. This card introduces the terminology of sensory attributes, explains the tasting procedure, and provides guidance on the type of scale used for assessment.

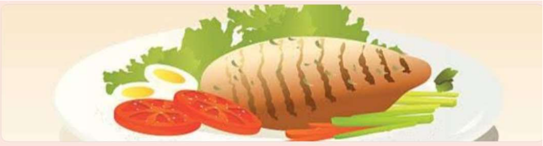

SURVEY

Answer all the questions

* Mandatory

Name and surname *

Judge's age *

- ◯ 18-25
- ◯ 26-35
- ◯ 36-45
- ◯ 46-55
- ◯ 56-65
- ◯ Over 65

GENDER *

- ◯ Female
- ◯ Male

Meat consumption per week *

- ◯ Never
- ◯ Several times a week
- ◯ Twice a week
- ◯ Once a week
- ◯ Other:

Poultry consumption per week (chicken, turkey)? *

- ◯ Several times a week
- ◯ Twice a week
- ◯ Once a week
- ◯ Other:
- ◯ Never

Cooking methods most frequently used for meat preparation *

Single or multiple choice

- ☐ Grill/Embers
- ☐ Pan-fried
- ☐ Steamed
- ☐ Boiled

Which sensory aspect should be prioritized to enhance the taste of meat? *

- ◯ Apparеance
- ◯ Tenderness, Juiciness, Chewiness
- ◯ Flavor
- ◯ Smell
- ◯ Other:

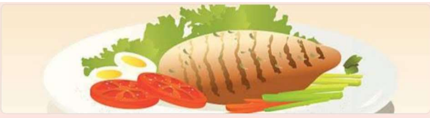

Sample (CODE)

IMPORTANT INFORMATION

Samples
and standards must be stored on the top shelf of the fridge at 4 °C.

Samples
must be individually prepared by heating them in a microwave oven (30 sec at 600 W), then removed from the package and finally tasted.

Please, taste samples in the requested order. Between chicken samples, please cleanse your mouth with a piece of cracker and some water.

* Mandatory

APPEARANCE: visual

DONENESS *
Definition: Degree of cooking.

Evaluation: please evalute each sample under white light. It goes from pink or light pink (when it is little cooked or no cooked at all) to white (when it is well cooked).

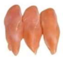
No cooked

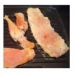
Little cooked

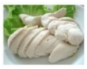
Cooked

	1	2	3	4	5	6	7	8	9	10	
Little cooked	○	○	○	○	○	○	○	○	○	○	Cooked

OLFACTORY PERCEPTION
Olfactory and flavor perceptions that remember smell and flavor of boiled meat, as well as chicken-like meat, perceived through nose and mouth.

BOILED MEAT SMELL *
Definition: characteristic odor released when meat is boiled, resulting from breakdown of proteins and fats during cooking.

Evaluation: place each sample approximately 1 cm under the nose and break it. Take 2–3 inhalations and rate the intensity of the boiled meat smell on a scale from 1 (very weak) to 10 (very strong).

	1	2	3	4	5	6	7	8	9	10	
Very weak	○	○	○	○	○	○	○	○	○	○	Very strong

LIKE-CHICKEN COOKED MEAT SMELL *
Definition: characteristic smell produced when meat is cooked, which resembles the familiar scent of cooked chicken.

Evalution: place the sample under the nose (1 cm) and brake it. Take 2-3 inhalations and rate the intensity of like-chicken cooked meat smell in on a scale from 1 (very weak) to 10 (very strong).

	1	2	3	4	5	6	7	8	9	10	
Very weak	○	○	○	○	○	○	○	○	○	○	Very strong

BOILED MEAT FLAVOR *
Definition: characteristic "aroma and taste" that occurs when meat is boiled, resulting from the breakdown of proteins, fats, and natural juices during cooking.

Evaluation: bring the sample to the mouth and chew it five times. Rate the intensity of the boiled meat flavor on a scale from 1 (very weak) to 10 (very strong).

	1	2	3	4	5	6	7	8	9	10	
Very weak	○	○	○	○	○	○	○	○	○	○	Very strong

LIKE-CHICKEN COOKED MEAT FLAVOR *
Definition: characteristic "aroma and taste" produced when meat is cooked which resembles the familiar flavor of cooked chicken.

Evaluation: bring the sample to the mouth and chew it five times. Rate the intensity of like-chicken cooked meat flavor on a scale from 1 (very weak) to 10 (very strong).

	1	2	3	4	5	6	7	8	9	10	
Very weak	○	○	○	○	○	○	○	○	○	○	Very strong

OTHER PERCEPTIONS *
Definition: perceptions such as bitter, sour, metallic, and astringent.

Evaluation: bring the sample to the mouth, chew it five times, and rate the intensity of 'other perceptions' of the cooked chicken meat, such as bitter, sour, metallic, or astringent sensations, on a scale from 1 (very weak) to 10 (very strong).

	1	2	3	4	5	6	7	8	9	10	
Very weak	○	○	○	○	○	○	○	○	○	○	Very strong

TEXTURE
The texture of meat refers to the sensory characteristics perceived during chewing, such as tenderness, juiciness, and chewiness. It depends on factors like the type of muscle, the content of connective tissue and fat, and the cooking method used.

TENDERNESS AT FIRST BITE *
Definition: the easiness of cutting the meat with the first bite.

Evaluation: bring the sample between the teeth and evaluate the stress required to cut the meat as tenderness perceived. Rate the tenderness at first bite on a scale from 1 (very low) to 10 (very high).

	1	2	3	4	5	6	7	8	9	10	
Very low	○	○	○	○	○	○	○	○	○	○	Very high

TENDERNESS *
Definition: the easiness of chewing the meat.

Evaluation: Bring the sample in mouth, chew it five times, and rate the chewing easiness of the meat as tenderness perceived. Rate the tenderness on a scale from 1 (very low) to 10 (very high).

	1	2	3	4	5	6	7	8	9	10	
Very low	○	○	○	○	○	○	○	○	○	○	Very high

JUICINESS *
Definition: the sensory perception of meat juice released after five chews.

Evaluation: bring the sample in mouth, chew it five times, and rate the amount of juice released from the meat as perceived juiciness. Rate the juiciness on a scale from 1 (very low) to 10 (very high).

	1	2	3	4	5	6	7	8	9	10	
Very low	○	○	○	○	○	○	○	○	○	○	Very high

RESIDUES OF CHEWING *
Definition: the amount of meat left after chewing.

Evaluation: bring the sample in mouth and evaluate the amount of meat remaining when it is ready to be swallowed. Rate the intensity on a scale from 1 (very weak) to 10 (very strong).

	1	2	3	4	5	6	7	8	9	10	
Very low	○	○	○	○	○	○	○	○	○	○	Very high

FINAL JUICINESS *
Definition: the sensory perception of meat juice at the end of chewing.

Evaluation: bring the sample in mouth and chew it until it is ready to be swallowed. Evaluate the amount of juice perceived on a scale from 1 (very low) to 10 (very high).

	1	2	3	4	5	6	7	8	9	10	
Very low	○	○	○	○	○	○	○	○	○	○	Very high

CHEWINESS *
Definition: the sensory sensation at the end of chewing.

Evaluation: bring the sample in mouth and evaluate the number of chews to make the sample ready at the swallowing on a scale from 1 (very low) to 10 (very high).

	1	2	3	4	5	6	7	8	9	10	
Very low	○	○	○	○	○	○	○	○	○	○	Very high

COMMENTS/NOTES
Please indicate any comments about the evaluated sensory attributes that are important for defining meat quality but are not reported on this card, including comments on pleasantness
