# Supplementary figures and images for: Adapting Sensory Analysis to the Pandemic Era: Exploring “Remote Home Tasting” of Sous-Vide Chicken Breast for Research Continuity
Source: Foods. 2025 Feb 14;14(4):647. doi: 10.3390/foods14040647 (PMC11854824; doi:10.3390/foods14040647)

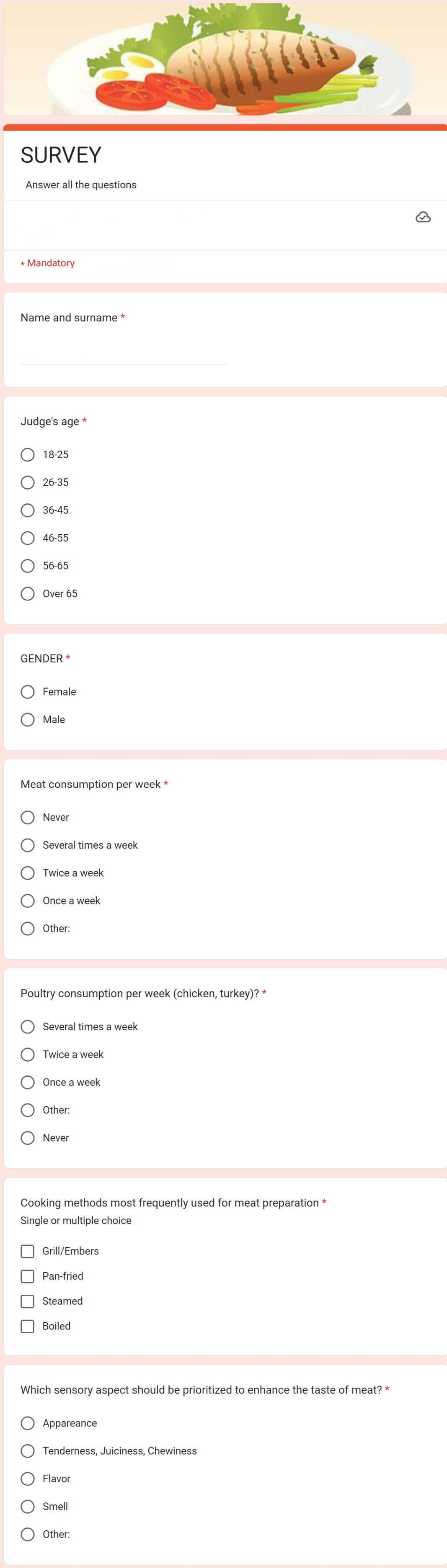

Supplement: Supplementary file 1 [file foods-14-00647-s001.zip › Fig._S1.tif]

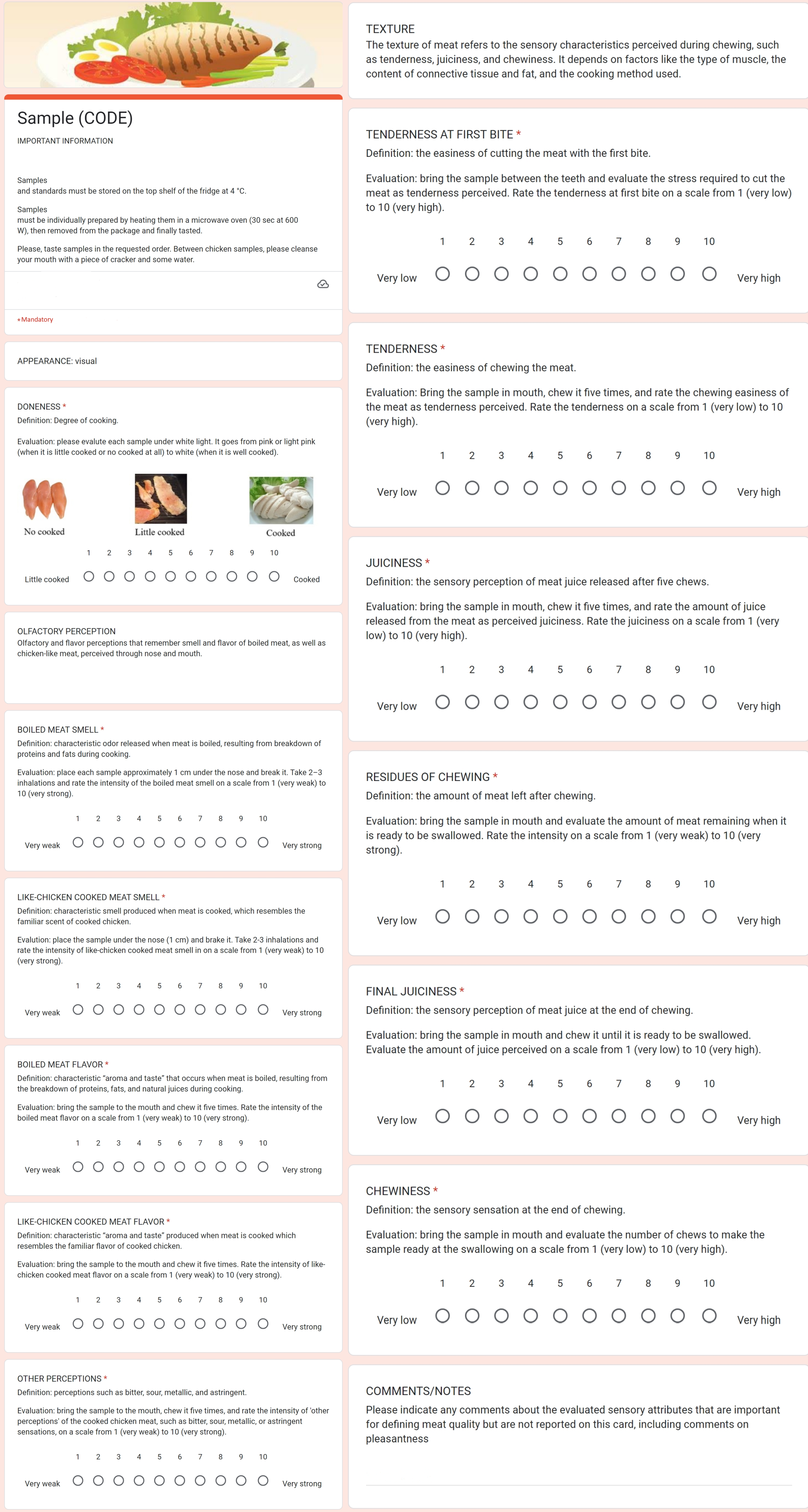

Supplement: Supplementary file 1 [file foods-14-00647-s001.zip › Fig._S2.tif]
